# Dominance of *Candidatus* Scalindua species in anammox community revealed in soils with different duration of rice paddy cultivation in Northeast China

**DOI:** 10.1007/s00253-012-4036-x

**Published:** 2012-04-19

**Authors:** Jing Wang, Ji-Dong Gu

**Affiliations:** 1 Laboratory of Environmental Microbiology and Toxicology, School of Biological Sciences, The University of Hong Kong, Pokfulam Road, Hong Kong SAR, People’s Republic of China; 2 The Swire Institute of Marine Science, The University of Hong Kong, Shek O, Cape d’Aguilar, Hong Kong SAR, People’s Republic of China

**Keywords:** Anammox bacteria, Rice paddy soil, 16S rRNA gene, Hydrazine oxidoreductase gene

## Abstract

The anaerobic ammonium-oxidizing (anammox) bacteria play an important role in the oxygen-limited zone for nitrogen cycling, but their roles in agricultural ecosystems are still poorly understood. In this study, soil samples were taken from the rhizosphere and non-rhizosphere and from surface (0–5 cm) and subsurface (20–25 cm) layers with 1, 4, and 9 years of rice cultivation history on the typical albic soil of Northeast China to examine the diversity and distribution of anammox bacteria based on 16S rRNA gene and hydrazine oxidoreductase encoding gene (*hzo*). By comparing these soil samples, no obvious difference was observed in community composition between the rhizosphere and non-rhizosphere or the surface and subsurface layers. Surprisingly, anammox bacterial communities of these rice paddy soils were consisted of mainly *Candidatus* Scalindua species, which are best known to be dominant in marine and pristine environments. The highest diversity was revealed in the 4-year paddy soil based on clone library analysis. Phylogenetic analysis of 16S rRNA gene and deduced HZO from the corresponding encoding gene showed that most of the obtained clones are grouped together with *Candidatus* Scalindua sorokinii, *Candidatus* Scalindua brodae, and *Candidatus* Scalindua spp. of seawater. The obtained clone sequences from all samples are distributed in two subclusters that contain sequences from environmental samples only. Tentative new species were also discovered in this paddy soil. This study provides the first evidence on the existence of anammox bacteria with limited diversity in agricultural ecosystems in Northern China.

**Electronic supplementary material:**

The online version of this article (doi:10.1007/s00253-012-4036-x) contains supplementary material, which is available to authorized users.

## Introduction

Nitrogen is the essential and structural component for proteins and is fundamental to structures and biochemical function for all living organisms (Jetten 2008). The fate of ammonium in the natural environments is of great importance for the microbial nitrogen cycle (Bothe et al. 2000; Strous and Jetten 2004; Schmid et al. 2007). Both ammonia-oxidizing bacteria and ammonia-oxidizing archaea have been long and recently known for their roles in oxidizing ammonia aerobically in a wide array of environments (Koops et al. 2003; Konneke et al. 2005; Cao et al. 2011a, b, c, d; 2012; Li et al. 2010a, b; 2011d). Furthermore, the anaerobic ammonium-oxidizing (anammox) process extends our understanding of the microbial diversity in the nitrogen cycle (Strous and Jetten 2004).

Anammox bacteria are autotrophic members belonging to the order *Planctomycetales*, which is a major distinct division of bacteria. Anammox bacteria are a group of slow-growing lithotrophic microorganisms (van de Graaf et al. 1996) and were firstly discovered to function in removing inorganic nitrogen from a wastewater treatment system (van de Graaf et al. 1995; Mulder et al. 1995; Jetten et al. 1997; Strous et al. 1997). Since then, a much wider distribution including oxygen minimum and oxygen-limited zones of oceans and marine sediments (Rysgaard et al. 2004; Dalsgaard et al. 2005; Hong et al. 2011b), sea water (Kuypers et al. 2005; Lam et al. 2007; van de Vossenberg et al. 2008), marsh (Koop-Jakobsen and Giblin 2009; Li et al. 2011a), wetland (Jetten et al. 2003; Zhu et al. 2010), estuary (Trimmer et al. 2003; Dale et al. 2009), freshwater ecosystem (Schubert et al. 2006; Rich et al. 2008), oil reservoir (Li et al. 2010a), and terrestrial ecosystem (Humbert et al. 2010) had been detected for the dominant process of anammox bacteria accounting for a significant portion of nitrogen loss.

As an artificial wetland, rice paddy soil is anoxic for the water-saturated sediment below the standing water (Li et al. 2009). As one of the most important agricultural activities and productive practices in human history (Liesack et al. 2000), paddy cultivation is still expanding to meet the demands of an increasing population. With large amount of nitrogen fertilizer applied in agriculture, in addition to the commonly recognized coupling nitrification and denitrification processes, the loss of nitrogen fertilizer from anaerobic soil can also be due to the anammox process known in many different ecosystems (Schubert et al. 2006; Francis et al. 2007; Schmid et al. 2007, 2008; Rich et al. 2008; Dale et al. 2009; Koop-Jakobsen and Giblin 2009; Cao et al. 2011a; Hong et al. 2011a, b; Li et al. 2011a, c).

The fully saturated paddy field during the whole growing season provides an ideal habitat for anammox bacteria, especially in the anoxic bulk soil according to in situ redox measurements (Noll et al. 2005). Much is now known about the nitrification and denitrification processes and many of the microorganisms involved in paddy sediments, but the newly discovered anammox bacteria have been rarely studied in an agricultural ecosystem, especially from paddy soil. In order to have a better understanding of the distribution and phylogenetic diversity of anammox bacteria in rice paddy field, particularly the vast area for agriculture with very low population density and short history of less than 60 years from wetland conversion to arable land, we collected both rhizosphere and non-rhizosphere paddy soil samples at different depths on Honghe State Farm of the Sanjiang Plain, Heilongjiang Province of Northeast China. Rice paddy fields under different years of cultivation history were selected, and the effects of soil management on anammox distribution were therefore evaluated.

## Materials and methods

### Sampling site

Honghe State Farm is situated on the Sanjiang Plain and was a natural wetland before converting into agricultural land after drainage in 1950s. Honghe Farm is characterized by its albic soil type, which is only found in northeast of China with high water holding capacity compared to the common agricultural soil type in this part of the country, e.g., black Chernozem soil. Albic soil is characterized by three distinguished layers of texture: a mollic epipedon, an albic E horizon, and a thick argillic horizon that is good for water holding but very poor for air flux vertically (Xing et al. 1994). In this study, sampling sites were randomly selected among the homogeneously managed farmland based on recorded history of cultivation and practices. Soybean was planted before the conversion into continuous rice paddy cultivation over the period in 1999–2008.

Samples were collected in late August 2008, 1 month before the harvest. Paddy soils from 1-year paddy field (changed into paddy cultivation in 2008), 4-year paddy field (changed into paddy cultivation back in 2005), and 9-year paddy field (changed into paddy cultivation in 2000) were collected from both rhizosphere and bulk soil for the surface (0–5 cm) and subsurface (20–25 cm) layers (bottom of the albic E horizon). Soil cores were randomly collected at four quadrat corners and one center point of each field using a soil core sampler, and the five samples of each specific depth were then homogenized, put in sterile plastic bags, and then transported back to the laboratory on dry ice in a cooler. They were stored at −20 °C in a refrigerator before further treatments and analysis. The pH value and oxidation–reduction potential (ORP) of the soils were measured in situ and detailed information is shown in Table 1.

**Table 1:** Sampling sites and sample description

Sample ID	Location	Sample description	Position to rice roots	Sampling depth (cm)	pH	ORP (mV)	Abbreviation^a^
S10	N 47°42′34.6″, E 113°31′01.3″	9 years under continuous paddy cultivation	Non-rhizosphere	0–5	5.01	−91.1	S10-9SN
S11			Non-rhizosphere	20–25	4.73	−100	S11-9BN
S12			Rhizosphere	0–5	6.76	−205.3	S12-9SR
S13			Rhizosphere	20–25	6.20	−172.7	S13-9BR
S14	N 47°43′13.2″, E 113°31′14.3″	4 years under continuous paddy cultivation	Non-rhizosphere	0–5	5.72	77	S14-4SN
S15			Non-rhizosphere	20–25	5.99	−184.4	S15-4BN
S16			Rhizosphere	0–5	5.73	−143.2	S16-4SR
S17			Rhizosphere	20–25	5.89	−2.8	S17-4BR
S18	N 47°43′34.9″, E 113°31′13.4″	1 year under paddy cultivation	Non-rhizosphere	0–5	6.34	−150.4	S18-1SN
S19			Rhizosphere	0–5	6.69	−176.6	S19-1SR
S20			Rhizosphere	20–25	6.65	−178.5	S20-1BR

^a^S10–S20 = sample ID, followed by years of paddy cultivation; S = surface sample (0–5 cm); B = bottom sample (20–25 cm); R = rhizosphere sample; N = non-rhizosphere sample

### DNA extraction

Approximately 0.6 g (wet weight) soil was transferred aseptically into a 1.5-ml screw-cap centrifuge tube. Total genomic DNA was extracted using Power Soil DNA Isolation Kit (Mo Bio, Carlsbad, CA, USA) according to the manufacturer’s instructions. Dilution of extracted DNA was applied if necessary afterward. All DNA samples were stored at −20 °C for further analyses as described below.

### PCR amplification of 16S rRNA and *hzo* genes

Two sets of PCR primer pairs were used for the detection of 16S rRNA gene, Brod541F–Amx820R and Amx368F–Amx820R, targeting *Candidatus* Scalindua and other groups of anammox bacteria, respectively. Other PCR primer pairs for amplifying hydrazine oxidoreductase encoding *hzo* gene of anammox were also applied in this study, and a 600-bp fragment that was suitable for taxonomy analysis was successfully generated. Detailed information of the PCR primers used in this study is presented in Table 2.

**Table 2:** Primer sets used in this study for amplification of 16S rRNA and *hzo* genes

Targets	Primers	5′–3′	Fragment size (bp)	Reference
16S	Brod541F	GAGCACGTAGGTGGGTTTGT	279	Li et al. (2010a, b)
	Amx820R	AAAACCCCTCTACTTAGTGCCC		
	Brod541F	GAGCACGTAGGTGGGTTTGT	719	Dale et al. (2009)
	Brod1260R	GGATTCGCTTCACCTCTCGG		
Nest-16S	Amx368F	CCTTTCGGGCATTGCGAA	452	
	Amx820R	AAAACCCCTCTACTTAGTGCCC		
*hzo*	Ana-hzo1F	TGTGCATGGTCAATTGAAAG	600	This study
	hzocl1R2	ACTCCAGATRTGCTGACC		

PCR amplification was performed in a 25-μl reaction system, containing 0.25 μM of each primer, 0.5 U of DNA polymerase (Promega), 5 μl 10× GoTag® Flexi Buffer, 50 mM MgCl_2_ solution, 500 μM (PCR Nucleotide Mix, 10 mM each) deoxynucleotide triphosphate, and 2.5 μl of 0.1 % BSA, and 25 ng of sample DNA to a final volume of 25 μl. The concentration of MgCl_2_ was adjusted slightly when amplifying *hzo* gene with different samples to optimize the performance. Amplification was performed with the MJ-Research Peltier Thermal Cycler (PTC-200, USA). The thermal profile used for amplification of 16S rRNA gene was modified after Li et al. (2010a, b) and Rich et al. (2008), including 5 min at 94 °C, followed by 40 cycles of 45 s at 94 °C, 30 s at 57 °C and 1 min at 72 °C, and 7 min at 72 °C for the last extension.

Amplification of *hzo* gene was performed by a rump program, including 5 min at 94 °C first, followed by 15 cycles of 1 min at 94 °C, 45 s at 48 °C, 1 min at 72 °C, and then followed by 1 min at 94 °C, an increase of 0.5 °C in every cycle up to 65 °C for annealing, and then 1 min at 72 °C, followed by a final 10 min at 72 °C for the last extension.

### Construction of clone libraries

Clone libraries of 16S rRNA and *hzo* genes of anammox bacteria in each sample were constructed according to Weidner (1996) for analyzing the community structures. Briefly, after total DNA extraction and PCR amplification of the 16S rRNA and *hzo* genes, PCR products were verified for correct amplification by running on a 1 % agarose gel in 1× TAE buffer at 90 V for 30 min. Gel slices containing the target PCR products were excised with sterilized knife and then purified using Gel Advanced Gel Extraction System DNA/RNA Extraction Kit (EG2002, Viogene). The purified PCR products were confirmed for its size again by running on agarose gel before ligation into pMD 18-T Vector (D101A, TaKaRa, Dalian, People’s Republic of China) and then cloned into *Escherichia coli* DH5-α cells according to the modified transformation method developed by Mandel and Higa (1970). Clones were randomly picked from each clone library and verified for correct insertion of DNA fragment by PCR amplification with the universal primer set M13F (5′-GTTTCCCAGTCACGAC-3′) and M13R (5′-TCACA CAGGAAACAGCTATGAC-3′). PCR products of the positive clones were purified using a PCR Purification Kit (Qiagen, USA) and then sequenced by Tech Dragon Ltd (Hong Kong). DNA sequences were examined and edited using BioEdit (Tom Hall, North Carolina State University, NC, USA).

### Phylogenetic analysis

The sequences from clone libraries were compared for homology and closest relatives in GenBank using BLAST tool (http://www.ncbi.nlm.nih.gov) and confirmed by their identities. The most closely related affinities and additional reference sequences were retrieved and consequently aligned together with representative clones in CLUSTAL X (version 2.0.11.). Neighbor-joining trees were created with MEGA [version 4.1 (Beta 2)] (Kumar et al. 2008). Cluster stabilities were assessed by bootstrap analyses based on 1,000 replicates.

### Data analysis

Sequences were analyzed in DOTUR to define operation taxonomic units (OTUs; Schloss and Handelsman 2005), and Shannon, Simpson, and Chao indices were subsequently calculated. An online software (UniFrac, http://bmf2.colorado.edu/unifrac/) was used for conducting the principal coordinate analyses (PCoA) and Jackknife Environment Clusters analyses using phylogenetic information (Lozupone et al. 2006). Pearson and two-sample *t* test were applied when significance test was required.

### Sequence accession number

The 16S rRNA and *hzo* gene sequences were deposited in the GenBank nucleotide sequence database under the accession JF965466 to JF965488 and JF999870 to JF99995, respectively.

## Results

### Community composition of anammox bacteria

The species composition and diversity index of anammox bacteria were analyzed by clone library. Two sets of PCR primer targeting16S rRNA gene and one pair of primer targeting *hzo* gene were used. In total, 391 clones from primer set Amx541F–Amx820R, 225 clones from primer set Amx368F–Amx820R, and 223 clones from primer set Ana-hzo1F–hzocl1R2 were randomly selected from each clone library and then sequenced. Sequences were compared with the current existing information available in NCBI by BLAST to identify their affinities first, and related anammox bacterial sequences were selected for phylogenetic analysis. Details of species composition and diversity indices are shown in Table 3.

**Table 3:** Species composition and diversity of anammox bacteria

Samples		S10-9SN	S11-9BN	S12-9SR	S13-9BR	S14-4SN	S15-4BN	S16-4SR	S17-4BR	S18-1SN	S19-1SR	S20-1BR
Primer set (1)												
541–820	No. of clones sequenced	24	26	32	30	33	41	42	41	42	41	39
	No. of *Planctomycetes*	13	13	16	13	21	6	21	16	23	16	17
	Percentage of *Planctomycetes*	54.1 %	50.0 %	50.0 %	43.3 %	63.6 %	14.6 %	50.0 %	39.0 %	54.7 %	39.0 %	43.5 %
	No. of anammox	3	13	16	13	15	6	20	12	12	16	16
	Percentage of anammox	12.5 %	50.0 %	50.0 %	43.3 %	45.4 %	14.6 %	47.6 %	29.2 %	28.5 %	39.0 %	41.0 %
	No. of OTUs	2	5	6	2	4	2	5	3	4	6	4
	Shannon index	0.69	1.38	1.54	0.93	1.85	0.87	1.51	1.62	0.85	1.96	0.92
Primer set (2)												
368–820	No. of clones sequenced	36	11	24	28	11	12	30	16	15	28	14
	No. of *Planctomycetes*	35	10	24	28	10	12	4	16	15	26	14
	Percentage of *Planctomycetes*	97.2 %	90.9 %	100.0 %	100.0 %	90.9 %	100.0 %	13.3 %	100.0 %	100.0 %	92.8 %	100.0 %
	No. of anammox	0	0	12	9	2	1	1	10	3	23	6
	Percentage of anammox	0.0 %	0.0 %	50.0 %	32.1 %	18.1 %	8.3 %	3.3 %	62.5 %	20.0 %	82.1 %	42.8 %
	No. of OTUs	4	1	2	3	3	2	2	4	3	3	3
	Shannon index	0.83	0.00	0.69	0.74	0.60	0.29	0.56	1.25	0.63	0.43	0.88
	Simpson	0.54	1.00	0.48	0.53	0.65	0.83	0.50	0.26	0.64	0.78	0.42
	Chao	4	1	2	3	4	2	2	4	3	3	3
Primer set (3)												
*hzo*	No. of clones sequenced	13	–	27	27	19	22	9	27	24	31	24
	No. of OTUs	1	–	1	1	2	1	1	2	1	1	1
	Shannon index	0		0	0	0.2	0	0	0.35	0	0	0
	Simpson	1		1	1	0.89	1	1	0.79	1	1	1
	Chao	1		1	1	2	1	1	2	1	1	1

*–* no PCR amplification product in this sample, *OTU* operation taxonomic unit

The proportion of *Planctomycetes* and anammox bacteria varied among each clone library and also varied between the two sets of primers. The highest diversity index appeared in 4-year paddy soils based on *hzo* gene and primer set Amx368–820 in sample S17-4BR, while with the results obtained by primer set Brod541F–Amx820R, the highest index came from 1-year paddy sample S19-1SR. The primer specificity of the anammox 16S rRNA gene is quite low for paddy soil samples in that most of the sequenced clones do not belong to anammox bacteria at all, and some of the clones are *Planctomycetes* bacteria. The proportion of *Planctomycetes* varied from 14.6 to 63.6 % in each clone library generated from primer set Brod541F–Amx820R, while the percentage of anammox bacteria varied from 14.6 to 50.0 %. When using primer set Amx368–820, the portion of *Planctomycetes* varied from 13.3 to 100 % among each clone library, while the proportion of anammox varied from 0 to 82.1 %. The samples that contained a higher percentage of *Planctomycetes* were not necessarily having higher percentage of anammox bacteria. For primer set Brod541F–Amx820R, sample S14-4SN showed the highest *Planctomycetes* bacteria composition (63.6 %), but the highest anammox composition was from samples S11-9BN and S12-9SR with 50 %, while the differences between samples were not significant (*p* < 0.05, *t* test). Using primer set Amx368–820, the proportion of *Planctomycetes* among sequenced clones in each clone library increased obviously up to 100 % in 6 out of the 11 samples. And except sample S16-4SR, the percentage of *Planctomycetes* was above 90 % among all the clones sequenced, with a percentage of 82.4 % in sample S19-1SR of anammox bacteria identified by this set of primer. In general, the primer set Amx368–820 seemed more effective in capturing anammox bacteria compared to Brod541F–Amx820R for the rice paddy soils investigated in this study.

The results of *hzo* gene clone library were more specific in molecular detection of anammox bacteria in that all the sequences in the clone libraries were relevant anammox *hzo* gene sequences. The diversity of anammox was quite low in these paddy soils according to the results of 16S rRNA gene with two pairs of primer, but it was even lower by *hzo* gene with only 1 OTU identified in most of the samples at 99 % cut-off value of protein sequences identity. Samples S14-4SN and S17-4BR of 4-year paddy soil, in which 2 OTUs were obtained, were proposed to have higher anammox diversity compared to 1- and 9-year paddy soils. The number of clones sequenced in each library reached the minimum requirement according to rarefraction curve generated by DTOUR analysis (Supplement material Fig. S1) as well as the OTUs identified met the Chao index in all samples.

### Phylogenetic analysis of 16S rRNA and *hzo* genes

**Fig. 1:**
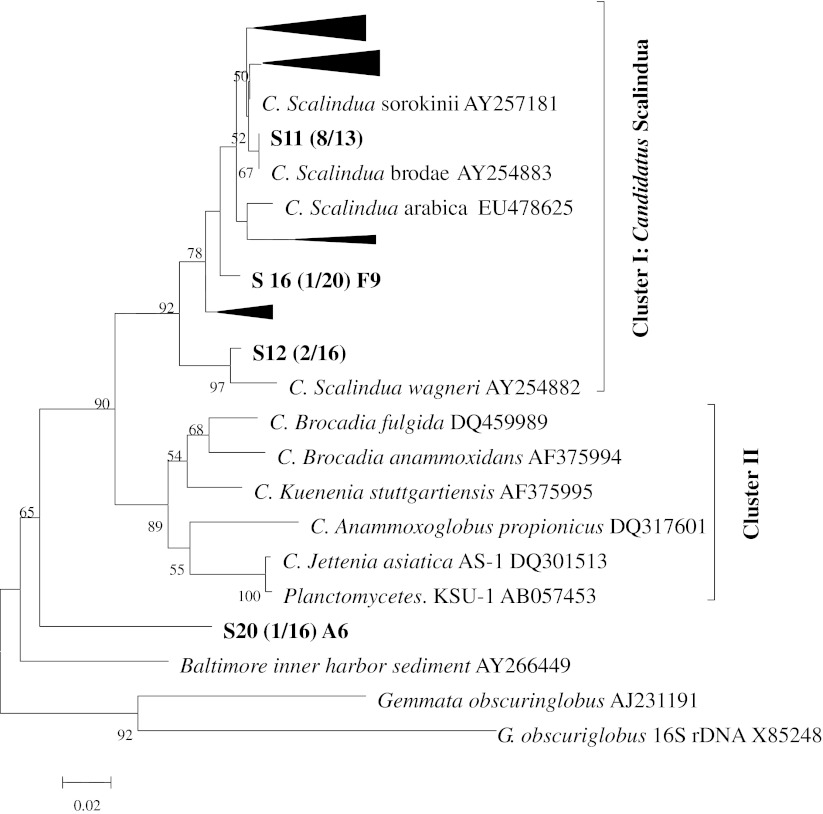
Neighbor-joining tree of phylogenetic analysis of 16S rRNA gene based on primer set Brod541F–Amx820R. *Numbers in parenthesis* indicate how many clones of that species were among all the anammox bacterial clones in the particular sample (*clones that included in the constrained groups, number of clones were included in the brackets followed after the sample name). (From *top* to *bottom*: *triangle 1*: S12 (3), S14 (1), S15 (1), S16 (2), S17 (1), S18 (1), S19 (2); *triangle 2*: S12 (7), S13 (7), S14 (10),S16 (13), S18 (1), S20 (11); *triangle 3*: S14 (1), S15 (1), S19 (3); *triangle 4*: S10 (3), S11 (5), S13 (2), S14 (3), S15 (4), S16 (3), S17 (11), S18 (1), S19 (9), S20 (4))

**Fig. 2:**
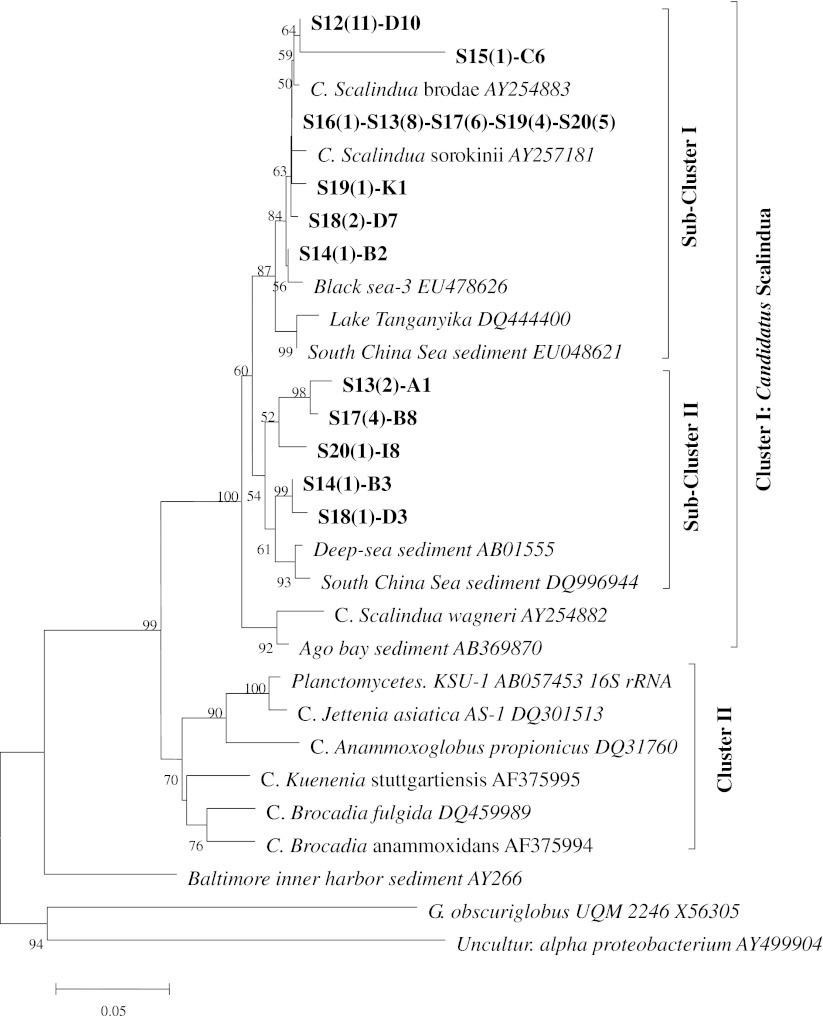
Neighbor-joining tree of phylogenetic analysis of 16S rRNA gene sequences based on primer set Amx368–820. The reference sequences are shown in italics, while the clones identified from this study are shown in *bold*. *Numbers in brackets* showed the quantity of clones belonging to that particular species. Bootstrap value was not shown when lower than 50

**Fig. 3:**
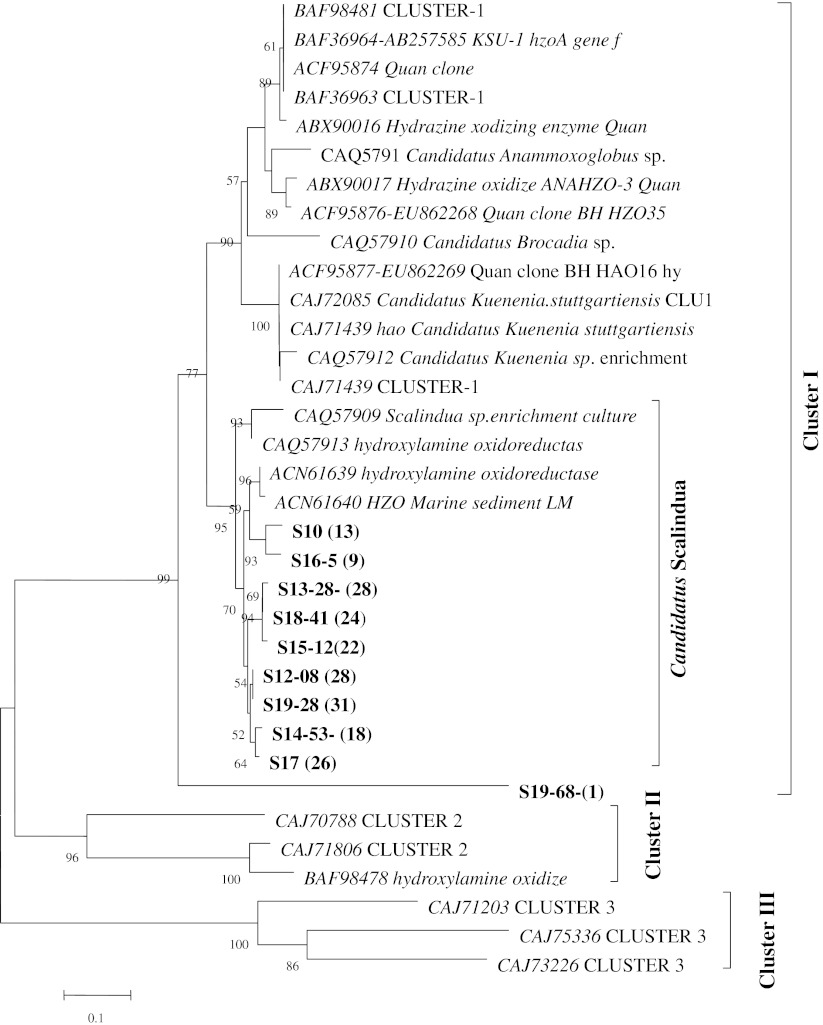
Neighbor-Joining tree showing the phylogenetic analysis of *hzo* gene and known anammox bacterial *hzo* gene based on deduced protein sequences. Clones from GenBank were shown in *italic*, and clones identified from this study were shown in *bold*. The *numbers in brackets* show the number of times a sequence was detected among all the tested clones of a sample. Bootstrap values (1,000 replicates) higher than 50 % were shown here and the *scale bar* represents 10 % of sequence divergence

Reference sequences in NCBI and closely related ones with more than 85 % similarity to the representative sequences obtained from this study were selected for phylogenetic analysis. All the sequences and their affinities were aligned and edited manually in CLUSTAL X before phylogenetic trees were constructed. Each single phylogenetic tree was constructed from the clone libraries generated by different primer sets separately and the results are shown in Figs. 1, 2 and 3.

Two distinct clusters can be distinguished in the phylogenetic tree generated by primer set Brod541F–Amx820R (Fig. 1). Cluster I contained only *Candidatus* Scalindua species, while the other three anammox genera together formed cluster II. All the sequences from this study were grouped into cluster I, except for one single clone from sample 20 which was out of the known anammox bacteria, but still included within *Planctomycetes* bacteria. In cluster I, two subclusters were identified. Two clones from sample 12 grouped with *Candidatus* Scalindua *wagneri* (AY254882), forming a small subcluster, and the rest of the anammox clones were grouped with *Candidatus* Scalindua sorokinii (AY257181), *Candidatus* Scalindua brodae (AY254883), and *Candidatus* Scalindua arabica (EU478625) to form a large subcluster. Since the similarity of the two clones from sample 12 was more than 85 % based on BLAST result, we propose that this sequence is a newly discovered anammox species from an agricultural environment, which is also supported by the high bootstrap values of 95 and 97 % in the phylogenetic tree.

In the neighbor-joining tree constructed based on primer set Amx368–820 (Fig. 2), where a longer 16S rRNA gene was amplified, anammox bacteria were also grouped into two subclusters within the large cluster of *Candidatus* Scalindua species. In subcluster I, similar to the Brod541F–Amx820R phylogenetic tree, most of the clones were closely related to the *Candidatus* Scalindua sorokinii (AY257181) and *Candidatus* Scalindua brodae (AY254883). In subcluster II, the clones were closely related to the environmental clones, mainly from deep sea sediments. No clone was falling outside the anammox cluster in the phylogenetic tree but sharing high similarity with *Planctomycetes* 16S rRNA sequences obtained with this pair of primer.

Quite a lot of *Acidomycetes* and rhizobules were detected by primer pair Brod541F–Amx820R. Although this primer set was proposed for all anammox bacteria from mangrove in Hong Kong (Li et al. 2011c), it was not specific enough for molecular detection of anammox bacteria in paddy soils of this study.

Similar to 16S rRNA analysis as shown in the neighbor-joining tree, the deduced Hzo protein sequences only showed one genus detected in our samples and it was included in cluster I (Fig. 3). No clone related to cluster II or cluster III could be detected in our samples. In addition, no clone related to other genera of anammox bacteria in cluster I was detected. The clones from all the samples were related to *Candidatus* Scalindua with more than 90 % similarity.

A difference was observed when comparing the results from 16S rRNA gene with those from the *hzo* gene: the former yielded more than one subclusters within the *Candidatus* Scalindua group, but the latter only formed a single branch closely related to *Candidatus* Scalindua spp. as well as some environmental clones from marine sediments. As an exception, one single clone, clone S19-68, was included within cluster I with a support of 99 % bootstrap value but formed a single branch among all the anammox *hzo* clones, suggesting a newly detected sequence from this rice paddy ecosystem.

Genera *Candidatus* Brocadia and *Candidatus* Kuenenia were traditionally found in wastewater treatment reactors or plants and their absence from these samples thus suggested that the sampling sites have not been affected significantly by anthropogenic sources of pollution and urbanization (Schmid et al. 2001; Dale et al. 2009; Cao et al. 2011a; Li et al. 2011c) because of low population density and less urbanization compared with coastal and central China.

### Correlation analysis

**Fig. 4:**
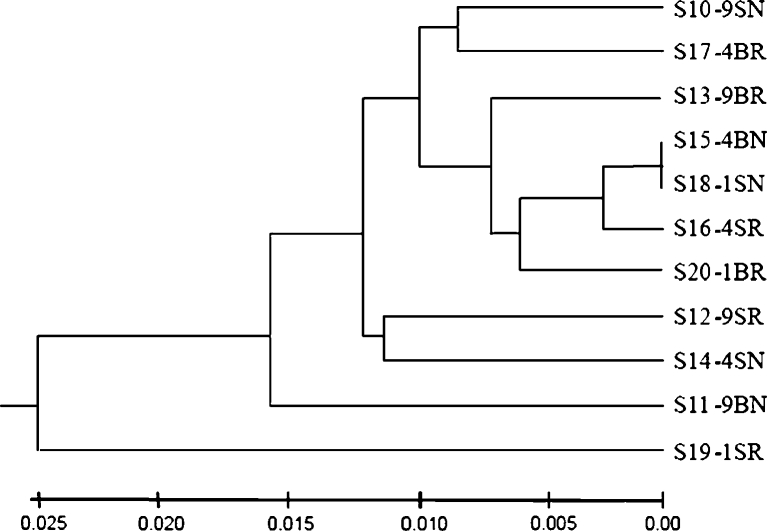
Dendrogram of the hierarchical clustering analysis of different characterized paddy samples of 16S rRNA gene sequences constructed by the UniFrac unweighted Jacknife Environment Clusters statistical method. Distances are shown in the *scale bar* below

**Fig. 5:**
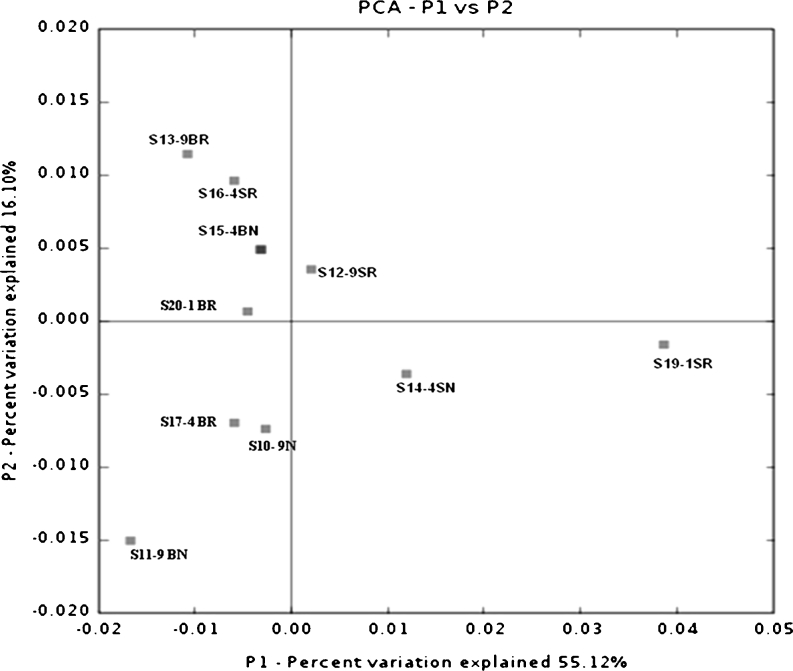
Ordination diagrams of paddy soil calculated with the unweighted UniFrac PCoA analysis using 16S rRNA gene sequences. Plot of the first two principal coordinate axes (P1 and P2) is shown here and the distributions of each assemblage (designated with the sample details) in response to the axes are shown on the plot

PCoA and UniFrac unweighted Jacknife environment cluster (Lozupone et al. 2007) were used for community analysis. A dendrogram of the 16S rRNA gene clustering analysis showed that two of the surface samples from the 1- and 4-year paddy soils grouped together first, but they were then grouped with subsurface samples from the same site hereafter. Also, 9-year paddy subsurface samples joined this big cluster, showing that there was no difference in community composition between different cultivation years of paddy field (Fig. 4), and the surface and subsurface layers did not show any obvious differences between them. The rhizosphere samples also grouped with non-rhizosphere samples as shown in clusters S10-9SN and S17-4BR, as well as clusters S12-9SR and S14-4SN. Among all the samples, S19-1SR was distinguished from the other samples. This result was further supported by PCoA analysis (Fig. 5). In this plot, three surface samples were separated from all the subsurface ones along the first principal coordinate (P1), but the samples from different years of paddy cultivation history did not show any difference along both the first and the second principal coordinates. The three non-rhizosphere samples were separated from all the rhizosphere samples along the second principal coordinate with 16.1 % of the variation explained among all the samples.

**Fig. 6:**
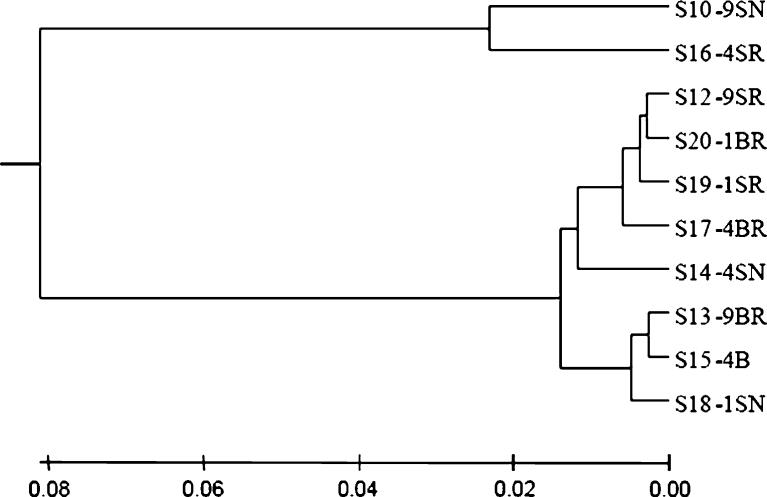
Dendrogram of the hierarchical clustering analysis of different characterized paddy samples *hzo* gene sequences constructed by the UniFrac unweighted Jacknife Environment Clusters statistical method. Distances were shown in the *scale bar* below

**Fig. 7:**
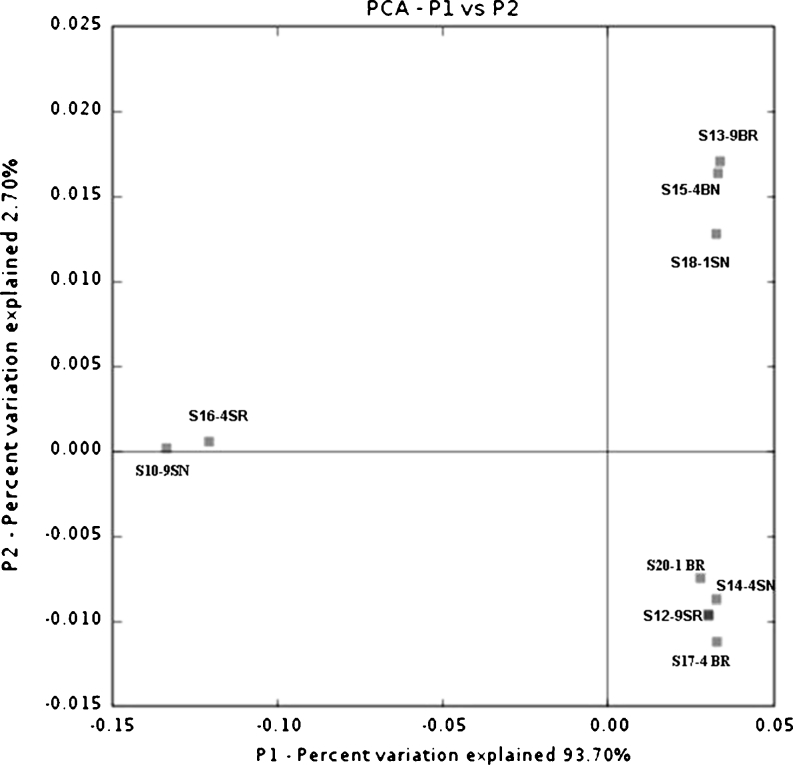
Ordination diagrams of paddy soil calculated with the unweighted UniFrac PCoA analysis using the *hzo* gene sequences. Plot of the first two principal coordinate axes (P1 and P2) is shown here and the distributions of each assemblage (designated with the sample details) in response to the axes are shown on the plot

In the dendrogram of clustering analysis generated by *hzo* gene sequences, different classifications were constructed (Fig. 6). The rhizosphere samples from different years and different layers were grouped together first, and then the non-rhizosphere samples were grouped with them. The two subsurface samples and two surface samples of 4 and 9 years, respectively, were grouped together, showing some similarity in community composition of the samples from these two different years. But 1-year samples were always grouped together with 4- and 9-year samples. In PCoA plot, this result was further supported by that no difference was observed in samples from different cultivation years (Fig. 7). Samples S10-9SN and S16-4SR were different from the others since they were distantly separated from the other samples along the first principal coordinate which explained 93.7 % variations among all the samples.

## Discussion

In this study, anammox bacteria were successfully detected from all rice paddy soil samples, which confirmed the research hypothesis that anammox bacteria are also potentially involved in the nitrogen loss from paddy soils in the albic soil of Northeast China the first time.

### Amplification of 16S rRNA gene

Due to the slow-growing rate, environmental detection of anammox bacteria has long been limited mostly to molecular techniques (Klotz and Stein 2008; Schalk et al. 2000; Shimamura et al. 2007; Li et al. 2010a; 2011a, b, c; Li and Gu 2011). Thereafter, the specificity of PCR primer set is critical for the successful and accurate identification of anammox bacteria from the environmental samples (Li et al. 2010b). Ever since the enrichment of anammox bacteria, 16S rRNA gene sequences of anammox bacteria have been made available in GenBank and could be used for the improvement of PCR primer design. Several primer sets targeting 16S rRNA gene of anammox bacteria have been designed and applied in wastewater treatment plants and sludge enrichment bioreactors (Schmid et al. 2000, 2003). Recently, new PCR primer sets had also been designed and applied to anammox detection in a wide range of environments (Neef et al. 1998), including estuarine, river sediment, terrestrial, and oil reservoirs (Dale et al. 2009; Humbert et al. 2010; Li et al. 2010a, Li et al. 2011a; Zhu et al. 2010).

Primer set Brod541F–Amx820R had been applied on marine and estuarine sediments for the detection of anammox bacteria, and their high specificity was confirmed with sediment samples from Mai Po mangrove sediment, mariculture zone, and the South China Sea (Li et al. 2011a, c; Hong et al. 2011b). This pair of PCR primer has been used for the detection of anammox bacteria in rice paddy soils for the first time, and good PCR products were obtained successfully and directly from all samples. However, subsequent results from the clone library showed that not all of the clones sequenced belonged to anammox or even *Planctomycetes* bacteria. High specificity of this pair of primer for mangrove and estuarine sediments was probably due to the relatively high abundance of anammox bacteria in those samples and dominant *Candidatus* Scalindua species, which are more similar to the anoxic marine sediment. The high divergence (<87.1 % similarity) among different genera of anammox bacteria makes detection by 16S rRNA gene-based PCR primer difficult when the biomass is very low (Jetten et al. 2009).

Primer set Amx368–820 had been applied for anammox detection in estuarine and a broad distribution of anammox bacteria was discovered (Dale et al. 2009). Although there was no visible PCR product observed in electrophoresis after the first round of PCR amplification, the second round nest PCR yielded satisfactory products from all samples similar to that reported in the study of Dale et al. It is reasonable that longer fragments would provide more information on taxonomy, but only one genus was detected by this pair of primer in this study. The low diversity of anammox bacteria in agricultural albic paddy soil in Northeast China is thought to be due to the short history of arable cultivation and also the clean groundwater used for irrigation in this part of China, which is very different from the extremely long history of human settlement in Southern China and also surface water for irrigation.

### Amplification of *hzo* gene

The hydrazine oxidoreductase (Hzo) encoded by *hzo* gene is a multi-heme protein abundantly produced by strain KSU-1 of an anammox bacterium (Shimamura et al. 2008). Hydrazine, an extremely toxic chemical compound, is an intermediate formed from coupling oxidation of ammonium and reduction of nitrite in the anammoxosome of anammox bacteria (Schalk et al. 2000). Hzo is one of the key enzymes involved in anammox reaction as it oxidizes the unique anammox transformation intermediate hydrazine to dinitrogen gas (N_2_) (Shimamura et al. 2007; Quan et al. 2008). For classification purposes, *hzo* is a functional gene that is more accurate and highly specific in classification and phylogeny compared to 16S rRNA gene (Li et al. 2010b). Detection of *hzo* gene was rarely reported from environmental samples due to the low efficiency of universal primer sets, but several attempts have been made recently in improving the efficiency of amplifying *hzo* genes (Li et al. 2010a, b; 2011a, c).

In order to have good reference sequences for phylogenetic analysis, currently published primer sets have been selected for analyzing the *hzo* gene in this study (Table 4). Unfortunately, no positive products have been amplified by any of the published primer pairs. Experimental work showed that amplification of *hzo* gene by the published primers is not as easy as 16S rRNA gene with environmental samples (Humbert et al. 2010). New combinations of these primer sets and also optimization of the PCR amplifying conditions according to the requirements of annealing temperature for different primer sets were made, and a positive PCR product of approximately 600 bp, which was included in cluster I of Hzo according to the published classification definition (Schmid et al. 2008), was obtained successfully by Ana-hzo1F and hzocl1R2. This is the first time for a successful amplification of *hzo* gene reported from rice paddy soil.

**Table 4:** Primer sets used for amplification of *hzo* gene

Taxonomy group	Primer pairs	5′–3′	Fragment size (bp)	Binding position relative to *hzo* CAJ72085	References
Cluster 1	hzocl1F1	TGYAAGACYTGYCAYTGG	470	739–757 and 1192–1209	Schmid et al. (2008)
	hzocl1R2	ACTCCAGATRTGCTGACC			
Cluster 1	hzocl1F1l	TGYAAGACYTGYCAYTGGG	470	739–758 and 1192–1209	Schmid et al. (2008)
	hzocl1R2	ACTCCAGATRTGCTGACC			
Cluster 2a	hzocl2aF1	GGTTGYCACACAAGGC	289	685–700 and 957–974	Schmid et al. (2008)
	hzocl2aR1	TYWACCTGGAACATACCC			
Cluster 2a	hzocl2aF1	GGTTGYCACACAAGGC	525	685–700 and 1506–1523	Schmid et al. (2008)
	hzocl2aR2	ATATTCACCATGYTTCCAG			
Cluster 2a	hzocl2aF2	GTTGTGMTGMWTGTCATGG	838	449-467 & 957-974	Schmid et al. (2008)
	hzocl2aR1	TYWACCTGGAACATACCC			
Cluster 1	Ana-hzo1F	TGTGCATGGTCAATTGAAAG	1,033	603–622 and 1636–1655	Quan et al. (2008)
	Ana-hzo2R	ACCTCTTCWGCAGGTGCAT			
Cluster 1	Ana-hzo1F	TGTGCATGGTCAATTGAAAG	600	603–622 and 1192–1209	This study
	hzocl1R2	ACTCCAGATRTGCTGACC			

The sequences obtained from primer sets of this study targeting Hzo cluster I could be grouped in good agreement with *Candidatus* Scalindua spp. as the deepest branch in the phylogenetic tree (Fig. 3). The efficiency of our primer set has also been tested by using samples from other habitats, such as marsh and wetland sediments, but no targeting product was obtained as smaller amplification segments often generated unexpectedly along with the rump program. Considering paddy soils, the specificity of primer set Ana-hzo1F and hzocl1R2 as a biomarker for a wider environmental detection of anammox needs to be further evaluated.

### Environmental detection of anammox bacteria

Since the first discovery and confirmation of anammox in wastewater treatment plants (van de Graaf et al. 1995), positive detection of anammox has been widely extended from laboratory systems to the large oxygen-limited area such as the ocean, and investigations into estuary and wetland ecosystems are also reported (Trimmer et al. 2003; Jetten et al. 2003; Dale et al. 2009; Li et al. 2011a; Zhu et al. 2010). But the artificial wetland, such as rice paddy field with the optimal conditions for anammox, had rarely been studied. High nitrogen input and active nitrogen loss from paddy field (Liesack et al. 2000; Hofstra and Bouwman 2005) are clear indications of the possible participation of such nitrogen transformation microorganisms. After a wide range of survey in terrestrial ecosystems, such as marshes, lakeshores, permafrost soil, agricultural soil as well as plant associated soil, Humbert et al. (2010) found that anammox bacteria are also widely distributed in these samples, and *Candidatus* Kuenenia and *Candidatus* Brocadia are the most common ones in the terrestrial ecosystem with human influences. Compared to the homogeneous marine water column environment, the diverse distribution of anammox bacteria in terrestrial ecosystem shows higher variations and adaption to different soils.

Both 16S rRNA and *hzo* genes could be applied to environmental samples and both of them showed abilities to separate niches from their own specific habitat, such as studies in marine, coastal, mangrove zones (Li et al. 2011a, c; Hong et al. 2011a), and oil reservoir (Li et al. 2010a). As a functional biomarker, *hzo* genes from environmental samples always reveal higher taxonomical accuracy and specificity of retrieved sequences, but their phylogenetic position has not been changed with different calculation methods employed (Klotz et al. 2008; Li et al. 2010a, b; Kartal et al. 2011). Growing evidence for the widespread occurrence of anammox in various ecosystems suggests the possibility that unknown anammox are yet to be discovered and investigated (Li et al. 2010a, b; Hong et al. 2011a). In this study, distinct clones revealed either by 16S rRNA gene or *hzo* gene are a strong indication for the detection of new species unique to this agricultural ecosystem.

### Anammox bacteria in paddy soil

Stable environmental conditions are thought to be a requirement for the harbor and enrichment of anammox bacteria in a natural environment (Dalsgaard et al. 2003, 2005; Humbert et al. 2010). The short agricultural cultivation history coupled with more recent rice paddy practice might be the major contributing factors limiting the diversity and distribution of anammox bacteria although they could be detected in all samples from the 1-year to 9-year paddy soils. Other than the history of paddy cultivation, the relative short history of land use from natural wetland to agricultural soil also should be considered when interpreting the occurrence of anammox bacteria obtained from Honghe farmland (Honghe nong chang 1986). This assumption was also supported by the successful detection of anammox bacteria in the Honghe wetland sediment (unpublished data), where the original wetland has been protected from human impact since.

Fertilization might have minor impact on the activity of anammox bacteria as no difference was observed in a marsh fertilization experiment (Koop-Jakobsen and Giblin 2009). The consistence and dominance of the genus *Candidatus* Scalindua in both Honghe wetland sediment and the rice paddy soil in Honghe farmland from this study further extends this conclusion. Unsuccessful amplification of anammox 16S rRNA and *hzo* genes from the soybean field of Honghe farmland (data not shown) indicates that flooding is the primary impact factor for the occurrence of anammox bacteria, though fertilization undoubtedly affects the activity of a whole range of nitrogen transformation microorganisms.

Previous investigations on anammox bacteria in natural environments suggest that it has a wide occurrence but low diversity in the anoxic marine sediment and sea water columns (Schmid et al. 2007). Moreover, both 16S rRNA and *hzo* genes reflect the same group of anammox that dominate the sea sediments (Hong et al. 2011a; Li et al. 2011a, c; Dang et al. 2010). The low diversity of anammox bacteria revealed by these two genes in paddy soil of this study is in good agreement with those reported previously. Our results showed an apparent dominance of *Candidatus* Scalindua spp. in every sample, providing further evidences for the niche specificity and distribution of the other four genera of anammox bacteria. In freshwater ecosystem, *Candidatus* Scalindua is always dominant, and in most cases, this is the only genus detected (Jetten et al. 2003). Though in the Cape Fear River estuary all four genera of anammox bacteria were detected by 16S rRNA gene, the possible pollution from adjacent wastewater treatment plant was argued to be the origin for the other three genera of anammox bacteria, which were commonly found in wastewater reactors before (Dale et al. 2009). In Honghe farmland, there is no potential pollution from adjacent areas because the population density is very low and there is no industry. The population density is 200,000 in an area of 12,400 km^2^. In addition, groundwater is used for irrigation, a much cleaner source than surface water. No clones belonging to *Candidatus* Kuenenia and *Candidatus* Brocadia were detected in any of the paddy soil samples of this study, but they were discovered from a paddy field in Southern China (Zhu et al. 2011), where anthropogenic impact on land is high with a long history of cultivation and much close proximity between human settlement and the agriculture land.

The activity of anammox can increase when the salinity decreased in the tidal marsh sediments (Koop-Jakobsen and Giblin 2009). It had been proposed that the occurrence of *Candidatus* Scalindua is highly related to the salinity level of the environment while *Candidatus* Brocadia and *Candidatus* Kuenenia are more abundant in low salinity environments, such as in estuarine and river sediments (Amano et al. 2007; Dale et al. 2009). The finding in this study sheds new light on the conventional belief on the environmental distribution of *Candidatus* Scalindua spp.

In paddy fields, only minimum oxygen gradient is available in soil along the vertical distribution section of a flooded area, as indicated from 140 μM in the water–soil interface to underdetection level at a depth of approximately 2.0 mm (Lüdemann et al. 2000). That might be the possible reason for no differences in both composition and distribution of anammox bacteria in the surface and subsurface layers of soil samples, as well as the rhizosphere and non-rhizosphere samples in this study.

In conclusion, anammox bacteria *Candidatus* Scalindua spp. were detected to be dominant in rice paddy soils in Sanjiang Plain of Northeast China with the highest diversity revealed in samples with 4-year cultivation history according to the combined 16S rRNA and *hzo* gene results. Soil samples from neither rhizosphere and non-rhizosphere nor surface and subsurface layers showed any habitat preference or impact on distribution of anammox bacteria in the rice paddy soils in this ecosystem. Retrieved DNA sequences also suggest new and unique ones indicating the possibility of new anammox species in this ecosystem.

## Electronic supplementary material

Below is the link to the electronic supplementary material.

ESM 1 (DOCX 75 kb)
