# Supplementary material for: Dominance of Candidatus Scalindua species in anammox community revealed in soils with different duration of rice paddy cultivation in Northeast China
Source: Appl Microbiol Biotechnol. 2012 Apr 19;97(4):1785–98. doi: 10.1007/s00253-012-4036-x (PMC3562551; doi:10.1007/s00253-012-4036-x)
Supplement: Supplementary file 1 — (DOCX 75 kb) [file 253_2012_4036_MOESM1_ESM.docx]

**Supplement Material**

Dominance of *Candidatus* Scalindua Species in Anammox Community Revealed in Soils with Different Duration of Paddy Rice Cultivation in Northeast China

## Jing Wang^1^, and Ji-Dong Gu^1,2^*

<1> Laboratory of Environmental Microbiology and Toxicology, School of Biological Sciences, The University of Hong Kong, Pokfulam Road, Hong Kong SAR, P.R. China

<2> The Swire Institute of Marine Science, The University of Hong Kong, Shek O, Cape d’Aguilar, Hong Kong SAR, P.R. China

***** Corresponding author: [jdgu@hkucc.hku.hk](mailto:jdgu@hkucc.hku.hk)

**Fig. S1** Rarefraction curves created using DOTUR based on AMX368–820 amplified 16S rRNA gene sequences in each clone library

No. of clones sequenced

**Fig. S1**
